# (+)-(*S*,*S*)-1,3-Bis[(tetra­hydro­furan-2-yl)­meth­yl]thio­urea

**DOI:** 10.1107/S1600536808040373

**Published:** 2008-12-13

**Authors:** Úlises Peña, Sylvain Bernès, René Gutiérrez

**Affiliations:** a Laboratorio de Síntesis de Complejos, Facultad de Ciencias Químicas, Universidad Autónoma de Puebla, AP 1067, 72001 Puebla, Pue., Mexico; b DEP Facultad de Ciencias Químicas, UANL, Guerrero y Progreso S/N, Col. Treviño, 64570 Monterrey, NL, Mexico

## Abstract

The title compound, C_11_H_20_N_2_O_2_S, is an enanti­omerically pure heterocycle-substituted thio­urea synthesized under solvent-free conditions. The thio­urea unit adopts a *ZZ* conformation, with the HN—(C=S)—NH core almost planar and the tetra­hydro­furfuryl groups placed below and above this plane. The whole mol­ecule thus approximates to noncrystallographic *C*_2_ symmetry. Unexpectedly, the C=S group is not involved in inter­molecular hydrogen bonding, as generally observed in homodisubstituted thioureas. Instead, mol­ecules form a one-dimensional network based on weak N—H⋯O(heterocycle) hydrogen bonding, resulting in a zigzag ribbon-like structure around the crystallographic 2_1_ screw axis along [100].

## Related literature

For general background about solvent-free synthesis, see: Tanaka & Toda (2000 ▶); Jeon *et al.* (2005 ▶). For *C*_2_ homosubstituted thio­ureas, see: Bailey *et al.* (1997 ▶); Lai & Tiekink (2002 ▶). For common hydrogen-bonding schemes in thio­ureas, see: Vázquez *et al.* (2004 ▶); Custelcean *et al.* (2005 ▶); Shashidhar *et al.* (2006 ▶); Sadiq-ur-Rehman *et al*. (2007 ▶); Saxena & Pike (2007 ▶).
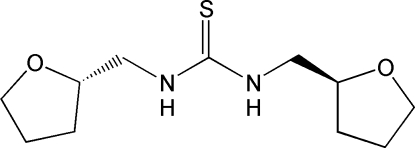


## Experimental

### 

#### Crystal data

C_11_H_20_N_2_O_2_S
*M* *_r_* = 244.35
Orthorhombic, 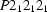

*a* = 7.8588 (9) Å
*b* = 10.8265 (11) Å
*c* = 15.6196 (16) Å
*V* = 1329.0 (2) Å^3^
*Z* = 4
Mo *K*α radiation
μ = 0.23 mm^−1^
*T* = 298 (1) K
0.6 × 0.6 × 0.6 mm

#### Data collection

Siemens P4 diffractometer
Absorption correction: ψ scan (*XSCANS*; Siemens, 1996 ▶) *T*_min_ = 0.782, *T*_max_ = 0.870
4611 measured reflections
3026 independent reflections
2484 reflections with *I* > 2σ(*I*)
*R*_int_ = 0.031
3 standard reflections every 97 reflections intensity decay: 1%

#### Refinement

*R*[*F* ^2^ > 2σ(*F* ^2^)] = 0.054
*wR*(*F* ^2^) = 0.165
*S* = 1.03
3026 reflections
151 parameters
2 restraints
H atoms treated by a mixture of independent and constrained refinement
Δρ_max_ = 0.22 e Å^−3^
Δρ_min_ = −0.16 e Å^−3^
Absolute structure: Flack (1983 ▶), 1267 Friedel pairs
Flack parameter: −0.01 (14)

### 

Data collection: *XSCANS* (Siemens, 1996 ▶); cell refinement: *XSCANS*; data reduction: *XSCANS*; program(s) used to solve structure: *SHELXS97* (Sheldrick, 2008 ▶); program(s) used to refine structure: *SHELXL97* (Sheldrick, 2008 ▶); molecular graphics: *Mercury* (Macrae *et al.*, 2006 ▶); software used to prepare material for publication: *SHELXL97*.

## Supplementary Material

Crystal structure: contains datablocks I, global. DOI: 10.1107/S1600536808040373/ci2734sup1.cif

Structure factors: contains datablocks I. DOI: 10.1107/S1600536808040373/ci2734Isup2.hkl

Additional supplementary materials:  crystallographic information; 3D view; checkCIF report

## Figures and Tables

**Table 1:** Hydrogen-bond geometry (Å, °)

*D*—H⋯*A*	*D*—H	H⋯*A*	*D*⋯*A*	*D*—H⋯*A*
N2—H2⋯O18^i^	0.85 (1)	2.095 (17)	2.897 (3)	157 (3)
N12—H12⋯O8^ii^	0.86 (1)	2.197 (18)	2.978 (3)	150 (3)

Symmetry codes: (i) 
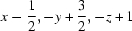
; (ii) 
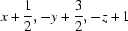
.

**Fig. 1.**
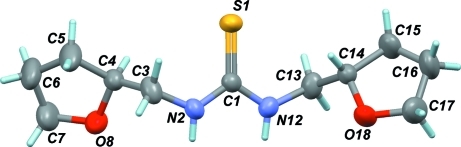
Molecular structure of the title compound, with 30% probability level displacement ellipsoids for non-H atoms.

**Fig. 2.**
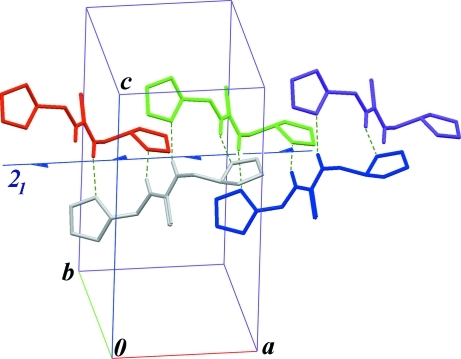
Part of the crystal structure of the title compound, showing the network of N—H···O hydrogen bonds (dashed lines). The 21 screw axis forming the backbone supramolecular structure is shown with a standard symbol, and each molecule has a color corresponding to symmetry related positions in the crystal: grey: asymmetric unit; red: -1/2 + x, 3/2 - y, 1 - z; green: 1/2 + x, 3/2 - y, 1 - z; blue: 1 + x, y, z; purple: 3/2 + x, 3/2 - y, 1 - z.

## supplementary crystallographic information

### Comment

The development of straightforward and eco-friendly synthetic procedures remains an important aim in organic synthesis. Many organic solvents, particularly chlorinated hydrocarbons, that are used in large quantities in organic reactions are potential threat to human health and environment. Thus, the design of chemical reactions under solvent-free conditions is getting a renewed interest. In this regard, solvent-free organic syntheses have great applied value and expansive prospects considering their advantages such as high efficiency and selectivity, easy separation and purification and environmental acceptability. All these merits are in accord with the green chemistry's requests of energy-saving, high efficiency and environmentally benign features (Tanaka & Toda, 2000; Jeon *et al.*, 2005). On the other hand, *N*,*N*'-disubstituted thioureas have recently received much interest due to their diverse applications, such as, *inter alia*, antiviral, antituberculous, fungicidal, herbicidal activities, as well as tranquilizing and antidiabetic drugs, agrochemical properties, antioxidants in gasoline, corrosion inhibitors, *etc*. In view of these and in continuation of our earlier work on the synthesis of thioureas (Vázquez *et al.*, 2004), we synthesized the title compound under solvent-free conditions (see *experimental*).

The asymmetric unit contains one molecule in general position (Fig. 1). As the amine used as starting material was enantiopure, the thiourea is found to be a pure (*S*,*S*) isomer. The central core HN—(C═S)—NH unit is close to be planar, the r.m.s. deviation from the mean plane S1/C1/N2/H2/N12/H12 being 0.039 Å. This core adopts a *ZZ* conformation (*i.e*. amine H atoms are arranged *syn*) and tetrahydrofurfuryl groups are placed below and above the central HN—(C═S)—NH plane. The whole molecule thus approximates a local *C*_2_ point symmetry. The observed conformation is identical to that found in other related homosubstituted thioureas (Lai & Tiekink, 2002; Bailey *et al.*, 1997).

The *ZZ* conformation avoids the formation of intramolecular hydrogen bonds (Saxena & Pike, 2007). Regarding the packing structure, it is clear that the thioketone functionality does not participate in intermolecular contacts. Such a situation is unexpected, since for previously X-ray characterized chiral and non-chiral homosubstituted thioureas, one-dimensional supramolecular structures based on C═S···H—N hydrogen bonds are predominant, providing that the thiourea is in a *ZZ* conformation (*e.g*. Vázquez *et al.*, 2004; Custelcean *et al.*, 2005; Shashidhar *et al.*, 2006; Sadiq-ur-Rehman *et al.*, 2007). Instead, the crystal structure of the title compound is determined by weak N—H···O(heterocycle) hydrogen bonds, aggregating molecules in a backbone arrangement (Fig. 2), parallel to the crystallographic 2_1_ screw axis along [100].

### Experimental

Under solvent-free conditions, (*S*)-(+)-tetrahydrofurfurylamine (0.49 g, 4.88 mmol) and CS_2_ (0.19 g, 2.44 mmol) were mixed at 298 K, giving a white solid. The crude was recrystallized from EtOH, affording colourless crystals of the title compound. Yield 99%; m.p. 376–378 K; [α]^25^_D_=+28.7 (*c*=1, CHCl_3_). Anal. Calcd for C_11_H_20_N_2_O_2_S: C 54.07, H 8.25, N 11.46, O 13.10, S 13.12%; found: C 53.12, H 8.18, N 11.30, O 12.98, S 13.87%. Spectroscopic data are in agreement with the X-ray formula (see archived CIF).

### Refinement

Methylene and methine H atoms were placed in idealized positions and refined as riding to their carrier C atoms. Amine H atoms, H2 and H12, were found in a difference map and refined with N—H bond lengths restrained to 0.86 (1) Å. For all H atoms, isotropic displacement parameters were calculated as *U*_iso_(H) = 1.2*U*_eq_(carrier atom).

### Crystal data

**Table:**

C_11_H_20_N_2_O_2_S	*D*_x_ = 1.221 Mg m^−^^3^
*M**_r_* = 244.35	Melting point = 376–378 K
Orthorhombic, *P*2_1_2_1_2_1_	Mo *K*α radiation, λ = 0.71073 Å
Hall symbol: P 2ac 2ab	Cell parameters from 78 reflections
*a* = 7.8588 (9) Å	θ = 4.6–13.7°
*b* = 10.8265 (11) Å	µ = 0.23 mm^−^^1^
*c* = 15.6196 (16) Å	*T* = 298 K
*V* = 1329.0 (2) Å^3^	Block, colourless
*Z* = 4	0.6 × 0.6 × 0.6 mm
*F*(000) = 528	

### Data collection

**Table:**

Siemens P4 diffractometer	2484 reflections with *I* > 2σ(*I*)
Radiation source: fine-focus sealed tube	*R*_int_ = 0.031
graphite	θ_max_ = 27.5°, θ_min_ = 2.3°
2θ/ω scans	*h* = −10→10
Absorption correction: ψ scan (*XSCANS*; Siemens, 1996)	*k* = −14→14
*T*_min_ = 0.782, *T*_max_ = 0.870	*l* = −20→20
4611 measured reflections	3 standard reflections every 97 reflections
3026 independent reflections	intensity decay: 1%

### Refinement

**Table:**

Refinement on *F*^2^	Secondary atom site location: difference Fourier map
Least-squares matrix: full	Hydrogen site location: inferred from neighbouring sites
*R*[*F*^2^ > 2σ(*F*^2^)] = 0.054	H atoms treated by a mixture of independent and constrained refinement
*wR*(*F*^2^) = 0.165	*w* = 1/[σ^2^(*F*_o_^2^) + (0.0916*P*)^2^ + 0.2186*P*] where *P* = (*F*_o_^2^ + 2*F*_c_^2^)/3
*S* = 1.03	(Δ/σ)_max_ = 0.001
3026 reflections	Δρ_max_ = 0.22 e Å^−^^3^
151 parameters	Δρ_min_ = −0.16 e Å^−^^3^
2 restraints	Absolute structure: Flack (1983), 1267 Friedel pairs
0 constraints	Flack parameter: −0.01 (14)
Primary atom site location: structure-invariant direct methods	

### Fractional atomic coordinates and isotropic or equivalent isotropic displacement parameters (Å^2^)

**Table:**

	*x*	*y*	*z*	*U*_iso_*/*U*_eq_	
S1	0.48959 (12)	0.46401 (7)	0.34087 (6)	0.0889 (3)	
C1	0.4852 (3)	0.5875 (2)	0.40545 (16)	0.0582 (5)	
N2	0.4051 (3)	0.6930 (2)	0.38410 (17)	0.0688 (6)	
H2	0.389 (5)	0.752 (2)	0.4186 (18)	0.083*	
C3	0.3199 (4)	0.7142 (4)	0.3037 (2)	0.0845 (9)	
H3A	0.3768	0.6668	0.2594	0.101*	
H3B	0.3303	0.8009	0.2889	0.101*	
C4	0.1361 (4)	0.6803 (3)	0.30448 (19)	0.0750 (8)	
H4A	0.1225	0.5952	0.3248	0.090*	
C5	0.0534 (7)	0.6947 (6)	0.2172 (3)	0.1303 (19)	
H5A	0.1088	0.7591	0.1842	0.156*	
H5B	0.0582	0.6180	0.1852	0.156*	
C6	−0.1224 (7)	0.7283 (7)	0.2368 (3)	0.146 (2)	
H6A	−0.1636	0.7898	0.1967	0.175*	
H6B	−0.1957	0.6563	0.2336	0.175*	
C7	−0.1204 (6)	0.7774 (6)	0.3230 (3)	0.1225 (16)	
H7A	−0.1498	0.8644	0.3220	0.147*	
H7B	−0.2033	0.7344	0.3580	0.147*	
O8	0.0435 (3)	0.7618 (2)	0.35753 (14)	0.0872 (7)	
N12	0.5602 (3)	0.5888 (2)	0.48261 (15)	0.0677 (5)	
H12	0.541 (4)	0.651 (2)	0.5153 (17)	0.081*	
C13	0.6525 (4)	0.4849 (3)	0.5184 (2)	0.0796 (8)	
H13A	0.5944	0.4092	0.5026	0.096*	
H13B	0.6503	0.4911	0.5803	0.096*	
C14	0.8356 (3)	0.4768 (2)	0.48913 (18)	0.0647 (6)	
H14A	0.8394	0.4820	0.4265	0.078*	
C15	0.9260 (6)	0.3588 (3)	0.5178 (4)	0.1075 (14)	
H15A	0.8723	0.3243	0.5684	0.129*	
H15B	0.9252	0.2972	0.4727	0.129*	
C16	1.0986 (6)	0.3991 (4)	0.5365 (4)	0.1293 (18)	
H16A	1.1421	0.3569	0.5867	0.155*	
H16B	1.1731	0.3815	0.4885	0.155*	
C17	1.0897 (5)	0.5297 (5)	0.5515 (4)	0.132 (2)	
H17A	1.1046	0.5465	0.6121	0.158*	
H17B	1.1801	0.5711	0.5205	0.158*	
O18	0.9296 (3)	0.57528 (19)	0.52400 (18)	0.0850 (7)	

### Atomic displacement parameters (Å^2^)

**Table:**

	*U*^11^	*U*^22^	*U*^33^	*U*^12^	*U*^13^	*U*^23^
S1	0.0894 (5)	0.0744 (4)	0.1029 (6)	0.0133 (4)	−0.0215 (5)	−0.0363 (4)
C1	0.0455 (10)	0.0558 (11)	0.0733 (14)	−0.0014 (10)	0.0034 (11)	−0.0067 (10)
N2	0.0585 (12)	0.0615 (12)	0.0864 (15)	0.0100 (10)	−0.0053 (11)	−0.0065 (11)
C3	0.0738 (18)	0.098 (2)	0.0813 (18)	0.0236 (18)	0.0124 (15)	0.0169 (17)
C4	0.0749 (18)	0.0776 (17)	0.0724 (16)	0.0170 (15)	−0.0110 (14)	−0.0098 (14)
C5	0.117 (3)	0.194 (5)	0.081 (2)	0.061 (4)	−0.026 (2)	−0.034 (3)
C6	0.114 (4)	0.217 (6)	0.107 (3)	0.064 (4)	−0.045 (3)	−0.035 (4)
C7	0.075 (2)	0.160 (4)	0.133 (4)	0.036 (3)	−0.009 (2)	−0.030 (3)
O8	0.0645 (11)	0.1192 (17)	0.0778 (12)	0.0116 (11)	0.0004 (10)	−0.0262 (11)
N12	0.0551 (11)	0.0755 (13)	0.0725 (13)	−0.0009 (10)	−0.0071 (10)	−0.0105 (11)
C13	0.0672 (15)	0.0860 (19)	0.0856 (18)	−0.0126 (14)	−0.0081 (14)	0.0201 (16)
C14	0.0654 (13)	0.0590 (13)	0.0698 (14)	0.0061 (11)	−0.0113 (11)	−0.0025 (12)
C15	0.107 (3)	0.0628 (16)	0.153 (4)	0.0133 (17)	−0.039 (3)	0.004 (2)
C16	0.096 (3)	0.104 (3)	0.188 (5)	0.032 (2)	−0.051 (3)	−0.006 (3)
C17	0.0660 (19)	0.128 (4)	0.201 (5)	0.005 (2)	−0.030 (3)	−0.052 (4)
O18	0.0640 (11)	0.0632 (10)	0.1278 (18)	−0.0006 (9)	−0.0082 (12)	−0.0171 (11)

### Geometric parameters (Å, °)

**Table:**

S1—C1	1.675 (2)	C7—H7B	0.97
C1—N12	1.342 (3)	N12—C13	1.450 (4)
C1—N2	1.346 (3)	N12—H12	0.862 (10)
N2—C3	1.442 (4)	C13—C14	1.513 (4)
N2—H2	0.849 (10)	C13—H13A	0.97
C3—C4	1.490 (5)	C13—H13B	0.97
C3—H3A	0.97	C14—O18	1.407 (3)
C3—H3B	0.97	C14—C15	1.529 (4)
C4—O8	1.413 (4)	C14—H14A	0.98
C4—C5	1.519 (5)	C15—C16	1.454 (7)
C4—H4A	0.98	C15—H15A	0.97
C5—C6	1.461 (7)	C15—H15B	0.97
C5—H5A	0.97	C16—C17	1.436 (6)
C5—H5B	0.97	C16—H16A	0.97
C6—C7	1.447 (6)	C16—H16B	0.97
C6—H6A	0.97	C17—O18	1.418 (5)
C6—H6B	0.97	C17—H17A	0.97
C7—O8	1.407 (5)	C17—H17B	0.97
C7—H7A	0.97		
			
N12—C1—N2	114.8 (2)	C7—O8—C4	108.8 (3)
N12—C1—S1	122.69 (19)	C1—N12—C13	123.9 (2)
N2—C1—S1	122.5 (2)	C1—N12—H12	118 (2)
C1—N2—C3	124.6 (3)	C13—N12—H12	118 (2)
C1—N2—H2	124 (2)	N12—C13—C14	113.8 (2)
C3—N2—H2	111 (3)	N12—C13—H13A	108.8
N2—C3—C4	113.8 (3)	C14—C13—H13A	108.8
N2—C3—H3A	108.8	N12—C13—H13B	108.8
C4—C3—H3A	108.8	C14—C13—H13B	108.8
N2—C3—H3B	108.8	H13A—C13—H13B	107.7
C4—C3—H3B	108.8	O18—C14—C13	109.8 (2)
H3A—C3—H3B	107.7	O18—C14—C15	106.0 (2)
O8—C4—C3	110.5 (3)	C13—C14—C15	113.7 (3)
O8—C4—C5	104.0 (3)	O18—C14—H14A	109.1
C3—C4—C5	112.5 (4)	C13—C14—H14A	109.1
O8—C4—H4A	109.9	C15—C14—H14A	109.1
C3—C4—H4A	109.9	C16—C15—C14	104.0 (3)
C5—C4—H4A	109.9	C16—C15—H15A	111.0
C6—C5—C4	104.0 (4)	C14—C15—H15A	111.0
C6—C5—H5A	111.0	C16—C15—H15B	111.0
C4—C5—H5A	111.0	C14—C15—H15B	111.0
C6—C5—H5B	111.0	H15A—C15—H15B	109.0
C4—C5—H5B	111.0	C17—C16—C15	106.4 (4)
H5A—C5—H5B	109.0	C17—C16—H16A	110.4
C7—C6—C5	106.0 (4)	C15—C16—H16A	110.4
C7—C6—H6A	110.5	C17—C16—H16B	110.4
C5—C6—H6A	110.5	C15—C16—H16B	110.4
C7—C6—H6B	110.5	H16A—C16—H16B	108.6
C5—C6—H6B	110.5	O18—C17—C16	109.7 (4)
H6A—C6—H6B	108.7	O18—C17—H17A	109.7
O8—C7—C6	108.8 (3)	C16—C17—H17A	109.7
O8—C7—H7A	109.9	O18—C17—H17B	109.7
C6—C7—H7A	109.9	C16—C17—H17B	109.7
O8—C7—H7B	109.9	H17A—C17—H17B	108.2
C6—C7—H7B	109.9	C14—O18—C17	108.6 (3)
H7A—C7—H7B	108.3		
			
N12—C1—N2—C3	178.2 (3)	N2—C1—N12—C13	179.6 (2)
S1—C1—N2—C3	−2.2 (4)	S1—C1—N12—C13	0.0 (4)
C1—N2—C3—C4	91.0 (4)	C1—N12—C13—C14	84.0 (3)
N2—C3—C4—O8	68.5 (4)	N12—C13—C14—O18	68.7 (3)
N2—C3—C4—C5	−175.7 (3)	N12—C13—C14—C15	−172.8 (3)
O8—C4—C5—C6	−28.9 (6)	O18—C14—C15—C16	−22.6 (5)
C3—C4—C5—C6	−148.5 (5)	C13—C14—C15—C16	−143.3 (4)
C4—C5—C6—C7	21.4 (7)	C14—C15—C16—C17	21.4 (7)
C5—C6—C7—O8	−6.3 (7)	C15—C16—C17—O18	−13.3 (8)
C6—C7—O8—C4	−12.9 (6)	C13—C14—O18—C17	138.2 (4)
C3—C4—O8—C7	146.8 (4)	C15—C14—O18—C17	15.0 (5)
C5—C4—O8—C7	25.9 (5)	C16—C17—O18—C14	−1.6 (7)

### Hydrogen-bond geometry (Å, °)

**Table:**

*D*—H···*A*	*D*—H	H···*A*	*D*···*A*	*D*—H···*A*
N2—H2···O18^i^	0.85 (1)	2.10 (2)	2.897 (3)	157 (3)
N12—H12···O8^ii^	0.86 (1)	2.20 (2)	2.978 (3)	150 (3)

Symmetry codes: (i) *x*−1/2, −*y*+3/2, −*z*+1; (ii) *x*+1/2, −*y*+3/2, −*z*+1.

## References

[bb1] Bailey, P. J., Grant, K. J. & Parsons, S. (1997). *Acta Cryst.* C**53**, 247–248.

[bb2] Custelcean, R., Gorbunova, M. G. & Bonnesen, P. V. (2005). *Chem. Eur. J.* **11**, 1459–1466. 10.1002/chem.200400973 15651024

[bb3] Flack, H. D. (1983). *Acta Cryst.* A**39**, 876–881.

[bb4] Jeon, S.-J., Li, H. & Walsh, P. J. (2005). *J. Am. Chem. Soc.* **127**, 16416–16425. 10.1021/ja052200m 16305227

[bb5] Lai, C. S. & Tiekink, E. R. T. (2002). *Acta Cryst.* E**58**, o538–o539.

[bb6] Macrae, C. F., Edgington, P. R., McCabe, P., Pidcock, E., Shields, G. P., Taylor, R., Towler, M. & van de Streek, J. (2006). *J. Appl. Cryst.* **39**, 453–457.

[bb7] Sadiq-ur-Rehman, Ali, S. & Parvez, M. (2007). *Acta Cryst.* E**63**, o640–o641. 10.1107/S1600536809021904 PMC2969502 21582844

[bb8] Saxena, A. & Pike, R. D. (2007). *J. Chem. Crystallogr.* **37**, 755–764.

[bb9] Shashidhar, Thiruvenkatam, V., Shivashankar, S. A., Halli, M. B. & Guru Row, T. N. (2006). *Acta Cryst.* E**62**, o1518–o1519.

[bb10] Sheldrick, G. M. (2008). *Acta Cryst.* A**64**, 112–122. 10.1107/S0108767307043930 18156677

[bb11] Siemens (1996). *XSCANS* Siemens Analytical X-ray Instruments Inc., Madison, Wisconsin, USA.

[bb12] Tanaka, K. & Toda, F. (2000). *Chem. Rev.* **100**, 1025–1074. 10.1021/cr940089p 11749257

[bb13] Vázquez, J., Bernès, S., Reyes, Y., Moya, M., Sharma, P., Álvarez, C. & Gutiérrez, R. (2004). *Synthesis*, pp. 1955–1958.

